# miRNA125b Downregulation: A Review of the Novel Paradigm of Psoriasis Epigenetic Regulation

**DOI:** 10.7759/cureus.6798

**Published:** 2020-01-28

**Authors:** Kulachanya Suwanwongse, Nehad Shabarek

**Affiliations:** 1 Internal Medicine, Lincoln Medical Center, New York City, USA

**Keywords:** psoriasis, mirna, rna, molecular, review, epigenetic, pathogenesis, mechanism, skin, genetic

## Abstract

MicroRNAs (miRNAs) are small, non-protein coding ribonucleic acids (RNAs) that play a critical role in the regulation of gene expression. Change in miRNA expression has been identified in various diseases, including psoriasis. Our narrative review provides an updated overview of current research on miRNA125b and its role in psoriasis pathogenesis. We used the keywords “psoriasis, “microRNA,” “miRNA,” “miR,” and “stRNA” to identify and select studies in PubMed, CINAHL, and Scopus databases from inception to March 2018. The references cited by the retrieved literature were reviewed to broaden our search results. miRNA125b downregulation leads to aberrant proliferation and differentiation of keratinocytes, which is a primary pathology found in psoriasis. Understanding the epigenetic alteration of miRNA125b expression and its underlying molecular mechanisms will help in identifying pathogenesis, diagnosis, and possibly curative treatment of psoriasis in the future.

## Introduction and background

Psoriasis is a common chronic inflammatory skin disease with no curative treatment and contributes to high physical and psychological burdens. The prevalence of psoriasis ranged from 1% to 12% among different populations, while the average worldwide prevalence is 2% [1,2]. Psoriasis lesions show an intense proliferation and aberrant differentiation of keratinocytes along with the infiltration of inflammatory cells in the epidermis layer [3]. The etiology of psoriasis remains unclear, but it is thought to be complex multi-factorial factors linked to genetic susceptibility, an imbalance in an epigenetic network, and environmental factors. 

MicroRNAs (miRNAs) are endogenous, single-stranded, and small ribonucleic acids (RNAs), which are non-protein coding, but play crucial roles in the modulation of post-transcriptionally gene expression [4]. Aberrant miRNA regulations associated with an increased risk of developing several human diseases. Alterations in miRNA expression in psoriasis were first identified in 2007 [5]. Since then, several miRNA changes in psoriasis and their underlying molecular mechanism have been proposed [6]. A decrease in miRNA125b expression impairs healthy skin metabolisms, including stimulation of keratinocyte hyperproliferation and impaired differentiation of keratinocytes [7]. In this review, we will discuss the role of miRNA125b and its underlying molecular mechanisms in psoriasis and suggest the development of therapeutic methods based on this current knowledge.

## Review

**Figure 1:**
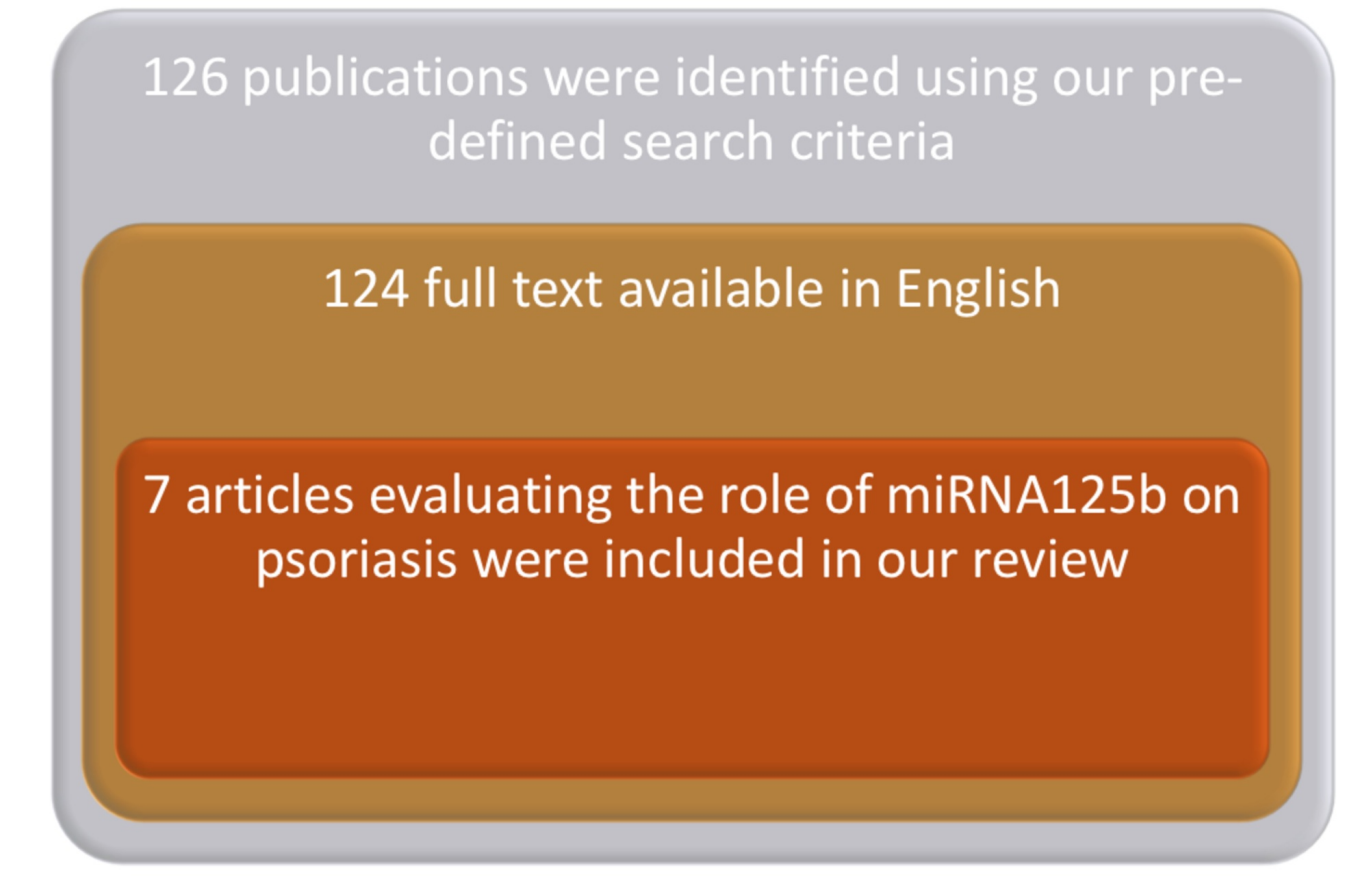
Concise PRISMA for included studies PRISMA, Preferred Reporting Items for Systematic Reviews and Meta-Analyses.

We conducted a narrative review of articles to evaluate the role of miRNA125b in psoriasis pathogenesis. Studies were searched and analyzed using keywords “psoriasis," “microRNA,” “miRNA,” “miR,” and “stRNA” in PubMed, CINAHL, and Scopus databases from inception to March 2018. The references cited by the retrieved literature were reviewed to broaden our search results. Relevant literature was included in this review as shown in Figure 1.

miRNAs are the most abundant class of human genome regulators, which account for 1% to 5% of the whole human genes and regulate up to 30% of protein-coding messenger RNAs (mRNAs) expression [8]. The primary function of miRNAs is to inhibit gene expression by binding to 3’ untranslated region of sequence-specific target mRNAs. The miRNA-mRNA complex then degrades, resulting in translational repression [8]. The inhibition of gene expression modulated via miRNAs sometimes cannot be seen in the post-transcriptional level, which means undetectable mRNA level change. Still, it is mostly detected in the post-translational level, demonstrated by detectable protein level change [9]. 

miRNA functions are comparable with transcription factors and considered as a part of epigenetic regulation. miRNAs regulate a broad range of cellular activities such as cell proliferation, development, and apoptosis [10]. Change of miRNAs expression has been identified in various diseases such as cancer and chronic inflammatory disorders, including psoriasis [10,11]. miRNAs mainly locate and function in cells. However, some miRNAs can be detected in an extracellular body fluid such as serum and urine [12,13]. Measurement of extracellular miRNAs helps in the diagnosis and evaluation of disease activity. Understanding the impact of alteration of miRNA expression on the pathogenesis of diseases will facilitate the development of therapeutic methods. For example, transfecting miRNAs to human pathologic cells may lead to cure for previously incurable diseases; also, the development of drugs to change miRNAs level in targeted cells may prevent the development of diseases altogether.

miRNA125b is one of the most miRNAs that showed the most dramatic change in its degree of expression in psoriasis. miRNA125b was found in most human organs; in the skin, miRNA125b is mostly expressed by resident cells, including keratinocytes, melanocytes, and fibroblasts [5]. The result from expression microarray and real-time quantitative reverse transcription-polymerase chain reaction showed that miRNA125b was significantly downregulated in psoriasis skin lesions, and the decrease in expression occurred mostly in keratinocytes [5,14]. 

miRNA125b is transcripted from two separated genes: pri-miRNA-125b-1 on chromosome 11, which is the primary precursor of miRNA125b in human skin, and pri-miRNA-125b-2 on chromosome 21 [15]. A decrease of pri-miRNA-125b-1 level was detected in psoriasis skin lesions so that the aberrant in miRNA125b expression should take place at the transcriptional level [14].

Xu et al. transfected human primary keratinocytes with miRNA125b precursor RNA (group 1) and miRNA125b inhibitor oligonucleotide (group 2) to evaluate the changes in their proliferation and differentiation. Group 1 keratinocytes showed a reduction in the proliferation rate, while group 2 showed an opposite result, i.e., an increase in proliferation rate [14]. This inverse correlation indicates that miRNA125b is a negative regulator of keratinocyte proliferation. In addition, keratinocyte differentiation markers, including involucrin, cytokeratin 10, filaggrin, and miRNA 203, were increased in group 1 and decreased in group 2 keratinocytes [14]. This forward correlation signified the role of miRNA125b as a promoter for keratinocyte differentiation.

The fibroblast growth factor receptor 2 (FGFR2) gene is a direct target of miRNA125b [14]. FGFR2 is a receptor on the epithelial cell membrane, and it plays a crucial role in regulating the growth and differentiation of epithelium [16]. A decrease in FGFR2 expression increased the keratinocyte proliferation rate. However, there was no correlation between keratinocyte differentiation and the level of FGFR2 expression [14]. Therefore, miRNA125b at least partially controls the keratinocyte proliferation by the FGFR2 pathway. In contrast, keratinocyte differentiation should be regulated by different miRNA125b-related pathways. FGFR2 was expressed primarily in stratum basalis, while miRNA125b expressed in all layers of the epidermis [14]. This discrepancy in spatial expression may point out the different underlying miRNA125b-related molecular mechanisms between keratinocytes in basal and suprabasal layers.

Ubiquitin-specific peptidase 2 (USP2) modulates TNF alpha-induced nuclear factor kappa B signaling and is another direct target of miRNA125b [4]. A decrease in USP2 expression reduces the keratinocyte proliferation rate and increases the keratinocyte differentiation, demonstrated by a rise in the mRNA expression level of differentiation markers (keratin 1410 and involucrin). Thus, miRNA125b regulates keratinocyte proliferation and differentiation, at least partly through the USP2-related pathway.

**Figure 2:**
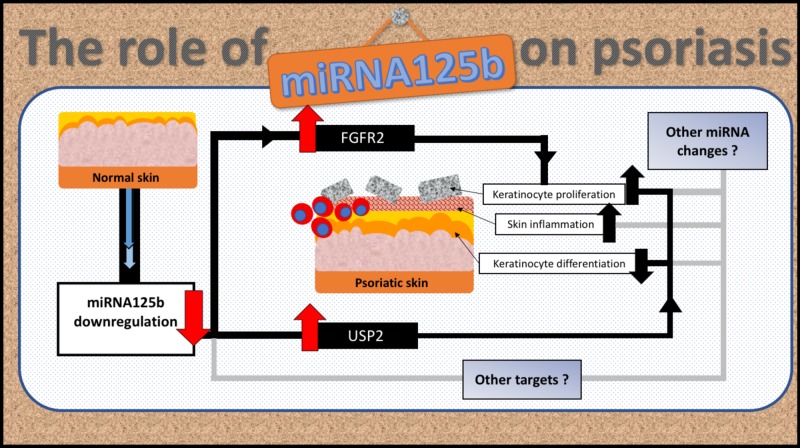
The underlying mechanisms of miRNA125b downregulation and the pathogenesis of psoriasis

Each miRNA has diverse functional roles in the human body. mi125b-related FGFR2 and USP2 pathways may contribute to only small arms for miRNA125b underlying cellular metabolism, as shown in Figure 2. In addition, the molecular pathogenesis of psoriasis does not depend solely on single miRNA aberration. Thus, there is a need to create a systematic link between each miRNA function and molecular tools to develop a comprehensive understanding of whole psoriasis pathogenesis. Nonetheless, understanding miRNA125b regulation pathways will help to formulate the underlying molecular mechanisms of psoriasis.

Although almost all studies, including this review, had good experimental designs, reliable method, and showed statistically significant results, they all had one limitation, that is, they are entirely conducted in vitro with the usage of several interference techniques. Functional experimental studies in vivo are required to demonstrate and validate the link between the alteration of miRNA125b expression and the underlying molecular mechanisms. Exploration of miRNA125b targeted genes will help to identify the key molecular changes in the development of psoriasis.

A decrease in the expression levels of miRNA125b is a characteristic of psoriasis and presumably contributes to its pathogenesis. The clinical application of miRNAs for the treatment of psoriasis should be considered. In theory, pathological downregulated miRNAs should be replaced by synthetic miRNAs, while an abnormal upregulated miRNA should be suppressed by using an miRNA inhibitor. The main advantage of treatment based on the miRNA method is that multiple factors causing the diseases can be treated at the same time. However, this is also a weakness because it will be problematic to create drug molecules with such board results without serious adverse effects. More research is needed to determine the impact of miRNA125 expression modification on the clinical course of psoriasis, the application of these fundamentals to psoriasis treatment, and the possible and prevention of the adverse effects that may occur. 

## Conclusions

Understanding the alteration of miRNA expression and their related molecular mechanisms will lead to more insights into pathogenesis, diagnosis, and treatment of psoriasis. miRNA125b was identified as a preventive factor for psoriasis by inhibiting proliferation and enhancing differentiation of keratinocytes. Further research determining the impact of alternating miRNA125b expression on the clinical course of psoriasis in vivo will provide more insight into psoriasis pathogenesis and possibly the development of treatment intervention. 
